# On the dimensionality of behavior

**DOI:** 10.1073/pnas.2021860119

**Published:** 2022-04-29

**Authors:** William Bialek

**Affiliations:** ^a^Joseph Henry Laboratories of Physics, Princeton University, Princeton, NJ 08544;; ^b^Lewis-Sigler Institute for Integrative Genomics, Princeton University, Princeton, NJ 08544;; ^c^Initiative for the Theoretical Sciences, The Graduate Center, City University of New York, New York, NY 10016

**Keywords:** information, prediction, complexity

## Abstract

There is a growing effort in the “physics of behavior” that aims at complete quantitative characterization of animal movements under more complex, naturalistic conditions. One reaction to the resulting explosion of high-dimensional data is the search for low-dimensional structure. Here I try to define more clearly what we mean by the dimensionality of behavior, where observable behavior may consist of either continuous trajectories or sequences of discrete states. This discussion also serves to isolate situations in which the dimensionality of behavior is effectively infinite.

## Significance

How do we characterize animal behavior? Psychophysics started with human behavior in the laboratory, and focused on simple contexts, such as the decision among just a few alternative actions in response to sensory inputs. In contrast, ethology focused on animal behavior in the natural environment, emphasizing that evolution selects potentially complex behaviors that are useful in specific contexts. New experimental methods now make it possible to monitor animal and human behaviors in vastly greater detail. This “physics of behavior” holds the promise of combining the psychophysicist’s quantitative approach with the ethologist’s appreciation of natural context. One question surrounding this growing body of data concerns the dimensionality of behavior. Here I try to give this concept a precise definition.

Observations on behavior provide a window into the dynamics of the brain and mind. This is an ancient idea, now receiving renewed attention because of the explosive growth of methods for quantitative measurements of behavior (1–8). These methods produce enormous quantities of raw data, such as high-resolution videos, so there is an obvious practical interest in data compression. This often involves searching for a low-dimensional description of the animal’s configuration or posture at each moment in time. This search is grounded both by the observation that even large and complex animals have relatively small numbers of muscles or joints and by direct evidence that motor behaviors are described by low-dimensional models in organisms from the worm *Caenorhabditis elegans* to humans and nonhuman primates (1, 9–14).

Reducing great volumes of video data to time series for just a few degrees of freedom is a triumph. The fact that this now can be done more or less automatically with machine learning methods means that exhaustive and quantitative characterization of behavior is possible in a much wider range of organisms, under a wider range of conditions. But the classical literature on dynamical systems reminds us that the time series of even a single variable could encode a higher-dimensional structure (15, 16). Indeed, this seems natural: The brain that generates behavior has many degrees of freedom, and observations of behavior should be sensitive to these potentially high-dimensional dynamics. On the other hand, the dynamics of large neural networks might be confined to low-dimensional manifolds, perhaps to match the dimensionality of motor behaviors (17–20).

All of these developments point to the problem of defining the dimensionality of behavior. In the extreme, we can imagine that the observable behavior reduces to a single function of time, as with the opening angle of a clamshell. Can we analyze this time series to identify a well-defined dimensionality for the underlying dynamics? Is it possible that this dimensionality is effectively infinite?

## A Context for Phenomenology

The quantitative analysis of behavior, including what follows here, is unapologetically phenomenological. The question is not “How does the brain generate behavior?” but rather “What is it about behavior that we would like to explain?” In an era of highly mechanistic biology, this emphasis on phenomenological description may seem odd. So, at the risk of repeating things that are well known, it is useful to remind ourselves of the long historical context for this approach.

If we want to explain why we look like our parents, a qualitative answer is that we carry copies of their DNA. But, if we want to understand the reliability with which traits pass from generation to generation, then DNA structure is not enough—the free energy differences between correct and incorrect base pairing are not sufficient to explain the reliability of molecular copying if the reactions are allowed to come to thermal equilibrium, and this problem arises not just in DNA replication but in every step of molecular information transmission. Cells achieve their observed reliability by holding these reactions away from equilibrium, allowing for proofreading or error correction (21, 22). In the absence of proofreading, the majority of proteins would contain at least one incorrect amino acid, and ∼10% of our genes would be different from those carried by either parent; these error rates are orders of magnitude larger than observed. These quantitative differences are so large that life without proofreading would be qualitatively different.*

The example of proofreading highlights the importance of starting with a quantitative characterization of the phenomena we are trying to explain. For brains and behavior, this is an old idea. In the late nineteenth century, many people were trying to turn observations on seeing and hearing into quantitative experiments, creating a subject that would come to be called psychophysics (23). By ∼1910, these experiments were sufficiently well developed that Lorentz could look at data on the “minimum visible” and suggest that the retina is capable of counting single photons (24), and Rayleigh could identify the conflict between our ability to localize low-frequency sounds and the conventional wisdom that we are “phase deaf” (25). Both of these essentially theoretical observations, grounded in quantitative descriptions of human behavior, drove experimental efforts that unfolded over more than half a century.

Also ∼1910, von Frisch (26) was doing psychophysics experiments to demonstrate bees could, in fact, discriminate among the beautiful colors of the flowers that they pollinate.^†^ But he took these experiments in a very different direction, focusing not on the discrete choices made by individual bees but on how these individuals communicated their sensory experiences to other residents of the hive, leading to the discovery of the “dance language” of bees. What grew out of the work by von Frisch and others was ethology (28), which emphasizes the richness of behavior in its natural context, the context in which it was selected for by evolution. Because ethologists wrestle with complex behaviors, they often resort to verbal description. In contrast, psychophysicists focus on situations in which subjects are constrained to a small number of discrete alternative behaviors, so it is natural to give a quantitative description by estimating the probabilities of different choices under various conditions.

The emergence of a quantitative language for the analysis of psychophysical experiments was aided by the focus on constrained behaviors, but was not an automatic consequence of this focus. For photon counting in vision, the underlying physics suggests how the probability of seeing vs. not seeing will depend on light intensity (29), but the observation that human observers behave as predicted points to profound facts about the underlying mechanisms (30). Attempts to formalize the problems of detection led to a more general view of the choices among discrete alternative behaviors being discriminations among signals in a background of noise (31), and, in the 1950s and 1960s, this view was exported to experimental psychology (23). Much of this now seems like an exercise in probability and statistics, something obviously correct, but the early literature records considerable skepticism about whether this (or perhaps any) mathematization of human behavior would succeed.

More generally, quantitative phenomenology has been foundational, certainly in physics and also in the mainstream of biology. Mendel’s genetics was a phenomenological description of the patterns of inheritance, and the realization that genes are arranged linearly along chromosomes came from a more refined quantitative analysis of these same patterns (32). The work of Hodgkin and Huxley (33) led to our modern understanding of electrical activity in terms of ion channel dynamics, but explicitly eschewed mechanistic claims in favor of phenomenology. The idea that transmission across a synapse depends on transmitter molecules packaged into vesicles emerged from the quantitative analysis of voltage fluctuations at the neuromuscular junction (34).

Even when we are searching for microscopic mechanisms, it is not anachronistic to explore macroscopic descriptions. Time and again, the scientific community has leaned on phenomenology to imagine the underlying mechanisms, often taking literally the individual terms in a mathematical description as representing the actual microscopic elements for which we should be searching, whether these are genes, ion channels, synaptic vesicles, or quarks (35–37). What is anachronistic, in the literal sense of the word, is to believe that microscopic mechanisms were discovered by direct microscopic observations without guidance from phenomenology on a larger scale.

In this broad context, how can we construct a quantitative phenomenology of complex, naturalistic behaviors? When we do psychophysics, we characterize behaviors with numbers that are meaningfully comparable across situations and across species. To give but one example, we can discuss the accumulation of evidence for decisions that humans and nonhuman primates make based on visual inputs, but we can use the same mathematical language to discuss decisions made by rodents based on auditory inputs (38). A quantitative characterization of naturalistic behaviors requires, similarly, that we attach comparable numbers to very different kinds of time series. The dimensionality of behavior is a candidate for this sort of unifying mathematical language.

## Two Examples

**Fig. 1.**
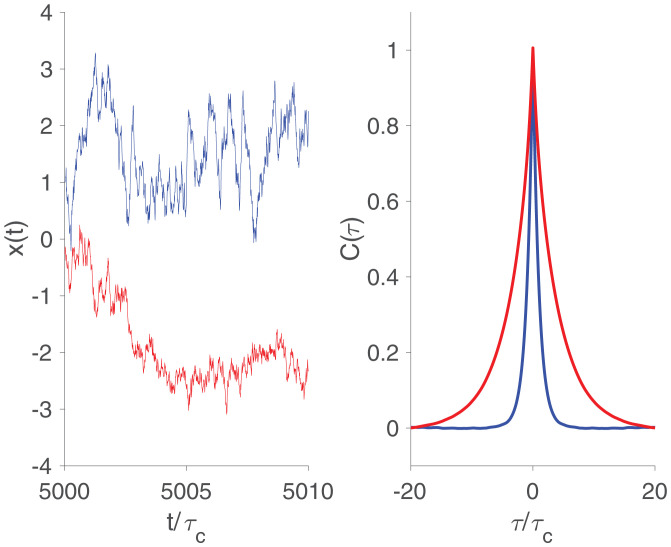
Two examples of behavioral trajectories (*Left*) and their correlation functions (*Right*). One-dimensional example, from Eq. **1**, is shown in blue. Two-dimensional example, from Eq. **4**, is shown in red. Behavioral trajectories are offset for clarity, and time is measured in units of the correlation time *τ_c_*.

To work toward a sharper definition, consider the case in which the behavior we observe is just a single function of time, *x*(*t*). Two examples of such trajectories are shown in Fig. 1, *Left*, and we will see that these correspond to one-dimensional (blue) and two-dimensional (red) systems. Qualitatively, the blue trajectory varies on one characteristic time scale, while the red trajectory involves rough movements on a short time scale superposed on smoother movements over a longer time scale. Our task is to make these observations precise.

Let’s work backward and start with a model for the behavior, a model in which it seems clear that the behavior really is one dimensional: The observed behavioral trajectory *x*(*t*) is described completely by

[1] $$\tau_{c}\frac{dx(t)}{dt}=-x(t)+\eta (t),$$

where η(t) is white noise,

[2] $$\langle \eta (t)\eta (t\prime )\rangle =2\tau_{c}\langle x^{2}\rangle \delta (t-t\prime ).$$

It is important that the noise source is white; nonwhite noise sources, which themselves are correlated over time, are equivalent to having hidden degrees of freedom that carry these correlations. The blue trajectory in Fig. 1, *Left* is drawn from a simulation of Eq. **1** with $\langle x^{2}\rangle =1$.

The observable consequences of the dynamics in Eqs. **1** and **2**. are well known: *x*(*t*) will be a Gaussian stochastic process, with the two-point correlation function

[3] $$C_{1}(\tau )=\langle x(t)x(t+\tau )\rangle =\langle x^{2}\rangle e^{-|\tau |/\tau_{c}},$$

shown in Fig. 1, *Right*. We recall that, for a Gaussian process, once we specify the two-point function, there is nothing else to say about the system. Importantly, we can turn this around: If the observed behavior is a Gaussian stochastic process, and the correlations decay exponentially as in Eq. **3**, then Eqs. **1** and **2** are a complete description of the dynamics.

An example of a clearly two-dimensional system involves not only the observable *x*(*t*) but also an internal variable *y*(*t*),

[4] $$\tau_{c}\frac{d}{dt}\left[\begin{array}{c} x(t)\\ y(t) \end{array}\right]=-\left[\begin{array}{cc} 1 & a\\ a & 1 \end{array}\right]\left[\begin{array}{c} x(t)\\ y(t) \end{array}\right]+\left[\begin{array}{c} \eta_{1}(t)\\ \eta_{2}(t) \end{array}\right].$$

To keep things simple, we can assume that the driving noises are independent of one another, and, again, they should be white so that we are not hiding additional variables that carry correlations. Since *y* is hidden, its units are arbitrary, which allows us to have the strength of the noise driving each variable be the same without loss of generality, so that

[5] $$\langle \eta_{\text{i}}(t)\eta_{\text{j}}(t\prime )\rangle =2\tau_{c}\langle x^{2}\rangle (1-a^{2})\delta_{\text{ij}}\delta (t-t\prime ).$$

The choice to give each variable the same correlation time is just for illustration, as is the symmetry of the dynamical matrix; the red trajectory in Fig. 1, *Left* is drawn from a simulation of Eq. **4** with $\langle x^{2}\rangle =1$ and a=0.75. Again, *x*(*t*) is Gaussian, but now the correlation function has two exponential decays,

[6] $$C_{2}(\tau )=A_{+}e^{-(1+a)|\tau |/\tau_{c}}+A_{-}e^{-(1-a)|\tau |/\tau_{c}}$$

[7] $$A_{\pm }=\frac{1}{2}\langle x^{2}\rangle \frac{\left(1-a^{2}\right)}{1\pm a},$$

shown in Fig. 1, *Right*. The short time scale $\tau_{c}/(1+a)$ corresponds to the rough movements seen in the trajectory, while $\tau_{c}/(1-a)$ corresponds to the smoother movements.

We see that a one-dimensional system generates behavior with a correlation function that has one exponential decay, while a two-dimensional system generates a correlation function with two exponential decays. We would like to turn this around, and say that, if we observe a certain structure in the behavioral correlations, then we can infer the underlying dimensionality.

## Gaussian Processes More Generally

Analyzing behavioral trajectories by constructing explicit dynamical equations, as in Eq. **1** or **4**, may not be the best approach. In particular, if there are hidden dimensions, then there is no preferred coordinate system in the space of unmeasured variables, and hence no unique form for the dynamical equations. Let us think, instead, about the probability distribution of the observed trajectories *x*(*t*). For Gaussian processes, this has the form

[8] $$P[x(t)]=\frac{1}{Z}e^{-S[x(t)]}$$

[9] $$S[x(t)]=\frac{1}{2}\int dt\int dt\prime \;x(t)K(t-t\prime )x(t\prime ),$$

where the integrals run over the interval of our observations, which should be long. The kernel K(τ) is inverse to the correlation function,

[10] ∫dt″ K(t−t″)〈x(t″)x(t′)〉=δ(t−t′).

We can divide the full trajectory *x*(*t*) into the past, $x_{\text{p}}$, with t≤0, and the future, $x_{\text{f}}$, with *t* > 0. Schematically,

[11] $$\begin{array}{l} S[x(t)]=\frac{1}{2}x_{\text{p}}\cdot K_{\text{pp}}\cdot x_{\text{p}}+\frac{1}{2}x_{\text{f}}\cdot K_{\text{ff}}\cdot x_{\text{f}}\\ \;\;\;\;+x_{\text{p}}\cdot K_{\text{pf}}\cdot x_{\text{f}}, \end{array}$$

where $K_{\text{pf}}$ couples the past and future. More explicitly,

[12] $$x_{\text{p}}\cdot K_{\text{pf}}\cdot x_{\text{f}}=\int_{0}^{\infty }dt\int_{0}^{\infty }dt\prime \;x(-t)K(t+t\prime )x(t\prime ).$$

If $K_{\text{pf}}$ is of finite rank, so that

[13] $$K(t+t\prime )=\sum_{\mu =1}^{D}a_{\mu }\phi_{\mu }(t)\phi_{\mu }(t\prime ),$$

then everything that we can predict about future behavior given knowledge of past behavior is captured by *D* features,

[14] $$P[x_{\text{f}}|x_{\text{p}}]=P[x_{\text{f}}|\{F_{\mu }\}]$$

[15] $$F_{\mu }=\int_{0}^{\infty }dt\;\phi_{\mu }(t)x(-t).$$

Eq. **14** is telling us that the features $\left\{F_{\mu }\right\}$ provide “sufficient statistics” for making predictions. We recall that, in a dynamical system with *D* variables,

[16] $$\frac{dy_{\text{i}}}{dt}=g_{\text{i}}(\{y_{\text{j}}\})+\eta_{\text{i}}(t),\;\;\;\;\;\text{i}=1,\;2,\;\cdots ,\;D,$$

predicting the future (*t* > 0) requires specifying *D* initial conditions (at *t* = 0). In this precise sense, the number of variables that we need to achieve maximum predictive power is the dimensionality of the dynamical system. To complete the argument, we need to show that $K_{\text{pf}}$ has finite rank when correlations decay as a finite combination of exponentials; see *Appendix A*.

**Fig. 2.**
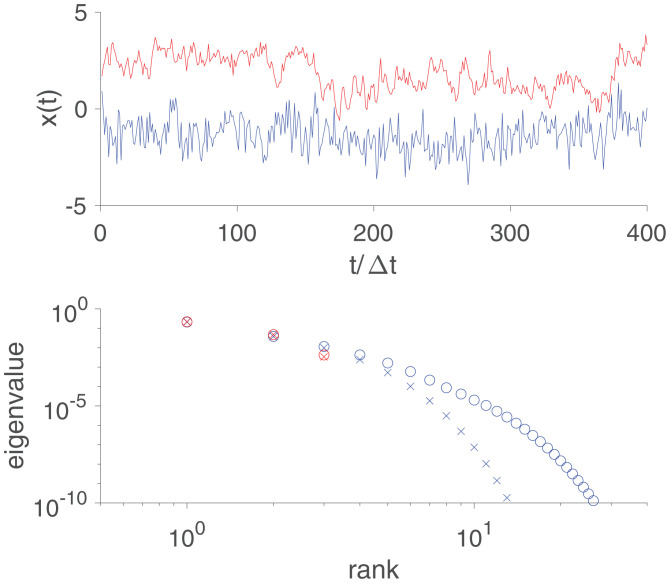
Sample trajectories (*Top*) and spectra of the matrix $K_{\text{pf}}$ (*Bottom*). In red is an example in which the underlying dynamics is three dimensional. In blue is an example with power law correlations, as in Eq. **17** with α=1/2, which is effectively infinite dimensional. Time is measured in discrete steps Δt, and spectra are computed in windows of duration T=100Δt (×) or T=1000Δt (°). Details are provided in *Appendix B*.

In the case of Gaussian stochastic processes, we thus arrive at a recipe for defining the dimensionality of the underlying dynamics. We estimate the correlation function, take its inverse to find the kernel, and isolate the part of this kernel which couples past and future. If this past–future kernel is of finite rank, then we can identify this rank with the dimensionality of the system. In Fig. 2, *Top* we see a sample trajectory (in red) from a system that is, by construction, three dimensional, with correlation times 4×,32×, and 256× the discrete time step (Δt) of our observations. As explained in *Appendix B*, the coefficients $a_{\mu }$ in Eq. **13** can be found as the eigenvalues of a symmetric matrix, and these eigenvalues are plotted in Fig. 2, *Bottom* in rank order (in red). This numerical analysis yields three clearly nonzero eigenvalues, with other eigenvalues below $10^{-10}$. Importantly, we find essentially the same three eigenvalues when the analysis is done in time windows of very different sizes—here T=100Δt and T=1,000Δt, smaller and larger, respectively, than the longest correlation time.

The past–future coupling is not guaranteed to be of finite rank. More generally, if we analyze signals in a window of size *T*, then the rank can grow with *T*. This happens, for example, if behavioral correlations decay as a power of time,

[17] $$\langle x(t)x(t\prime )\rangle =\langle x^{2}\rangle \frac{t_{0}^{\alpha }}{t_{0}^{\alpha }+|t-t\prime {|}^{\alpha }}.$$

Fig. 2, *Top* shows a sample trajectory from a Gaussian process with this correlation function (in blue), and Fig. 2, *Bottom* shows the associated spectrum of coefficients $a_{\mu }$ for α=1/2 (in blue). This illustrates both that there is no obvious cutoff to the spectrum and that the spectrum extends farther when the analysis is done in longer time windows [T=100Δt (×) vs. T=1000Δt (°)]. Indeed, the larger the window, the farther the spectrum extends, with no bound. Under these conditions, the dimensionality is effectively infinite.

The possibility that behavioral correlations decay as a power of time has a long and sometimes contentious history. It thus is worth noting that scaling of the correlation function implies an effectively infinite dimensionality, but it is not required. We can imagine situations in which the kernel $K_{\text{pf}}$ has an arbitrarily large number of nonzero eigenvalues in the limit of long observation times even if the correlation is not precisely a power law.

While the relation of dimensionality to the spectrum of $K_{\text{pf}}$ is attractive, estimating this spectrum from finite data can be challenging. Even if the true spectrum has only a finite number of nonzero eigenvalues, in matrices built from finite samples of data, the zero eigenvalues will be replaced by a continuous spectrum, and this could make it difficult, in practice, to distinguish finite from infinite dimensional processes. At the same time, it is important to emphasize that difficulty in resolving eigenvalues against a continuum generated by finite sample size is not evidence for low dimensionality, nor should a continuum be assigned as noise without further analysis. Random matrix theory provides quantitative predictions for spectral broadening in closely related contexts, including the dependence of spectra on sample size and matrix dimensionality, and these should provide a basis for identifying the contributions of noise to the observed eigenvalue spectra (39).

## Discrete States

In many cases, it is natural to describe animal behavior as moving through a sequence of discrete states. We do this, for example, when we transcribe human speech to text, and when we describe a bacterium as running or tumbling (40). This identification of discrete states is not just an arbitrary quantization of continuous motor outputs, nor should it be a qualitative judgment by human observers. Discrete states should correspond to distinguishable clusters, or resolvable peaks in the distribution over the natural continuous variables, and the dynamics should consist of movements in the neighborhood of one peak that are punctuated by relatively rapid jumps to another peak (e.g., ref. 3). A “mechanism” for such discreteness is the existence of multiple dynamical attractors, with jumps driven by noise (e.g., refs. 1 and 13).

When behavioral states are discrete, how do we define dimensionality? Once again, it is useful to think about the simplest case, where there are just two behavioral states—perhaps “doing something” and “doing nothing”—and time is marked by discrete ticks of a clock. We can represent the two states at each time *t* by an Ising variable $\sigma_{t}=\pm 1$. If the sequence of behavioral states were Markovian, then *σ_t_* depends only on $\sigma_{t-1}$, and, because $\sigma^{2}=1$, the only possible stationary probability distribution for the sequences $\sigma_{1},\;\sigma_{2},\;\cdots ,\;\sigma_{T}$ is

[18] $$P\left(\left\{\sigma_{t}\right\}\right)=\frac{1}{Z}\exp\;\left[h\sum_{t}\sigma_{t}+J\sum_{t}\sigma_{t-1}\sigma_{t}\right],$$

which is the one-dimensional Ising model with nearest-neighbor interactions. Importantly, if we measure the correlations of the fluctuations in behavioral state around its mean,

[19] $$C(t-t\prime )\equiv \langle \left(\sigma_{t}-\langle \sigma \rangle \right)\left(\sigma_{t\prime }-\langle \sigma \rangle \right)\rangle ,$$

we find that these correlations decay exponentially,

[20] $$C(t-t\prime )=C(0)e^{-|t-t\prime |/\tau_{c}},$$

where we can express *τ_c_* in terms of *h* and *J* (41). This reminds us of the exponential decays in the continuous case with Gaussian fluctuations.

Suppose that we have only two states, but observe correlations that do not decay as a single exponential. Then the probability distribution $P\left(\left\{\sigma_{t}\right\}\right)$ must have terms that describe explicit dependences of *σ_t_* on $\sigma_{t\prime }$ with t−t′>1. This can be true only if there are some hidden states or variables that carry memory across the temporal gap t−t′. A sensible definition for the dimensionality of behavior then refers to these internal variables.

Imagine that we observe the mean of the behavioral variable, 〈σ〉, and the correlation function C(t−t′). What can we say about the probability distribution $P\left(\left\{\sigma_{t}\right\}\right)$? There are infinitely many models that are consistent with measurements of just these (two-point) correlations, but there is one that stands out as having the minimal structure required to match these observations (42). Said another way, there is a unique model that predicts the observed correlations but otherwise generates behavioral sequences that are as random as possible. This minimally structured model is the one that has the largest possible entropy, and it has the form

[21] $$P\left(\left\{\sigma_{t}\right\}\right)=\frac{1}{Z}\exp\;\left[h\sum_{t}\sigma_{t}+\frac{1}{2}\sum_{t,t\prime }J(t-t\prime )\sigma_{t}\sigma_{t\prime }\right],$$

where the parameter *h* must be adjusted so that the model predicts the observed mean behavior 〈σ〉, and the function J(t−t′) must be adjusted so that the model predicts the observed correlation function C(t−t′).

Maximum entropy models have a long history, and a deep connection to statistical mechanics (42). As applied to temporal sequences, the maximum entropy models sometimes are referred to as maximum caliber (43). For biological systems, there has been interest in the use of maximum entropy methods to describe amino acid sequence variation in protein families (44–46), patterns of electrical activity in populations of neurons (47–51), velocity fluctuations in flocks of birds (52, 53), and more. There have been more limited attempts to use these ideas in describing temporal sequences, in neural populations (54) and in flocks (55–57).

To connect with the previous discussion, for continuous variables, a Gaussian process is the maximum entropy model consistent with the measured (two-point) correlations. In particular, if correlations decay as a combination of exponentials, then, in discrete time, the relevant Gaussian model has maximum entropy consistent with correlations among a finite number of neighboring time points. These models can also be written as autoregressive processes (58).

The maximum entropy model in Eq. **21** can be rewritten exactly as a model in which the behavioral state at time *t* depends only on some internal variable *x*(*t*). As explained in *Appendix C*, *x*(*t*) is not Gaussian, but the only coupling of past and future, again, is through a kernel *K*(*t*). This kernel is not the inverse of the observed behavioral correlations but of the effective interactions between states at different times, J(τ). But, importantly, we are considering quantities that are determined by the correlation function, and hence the problem is conceptually similar to the Gaussian case: We analyze the correlations to derive a kernel, and the dimensionality of behavior is the rank of this kernel. The maximum entropy model plays a useful role because it is the least structured model consistent with the observed correlations.

If *x*(*t*) is one dimensional in the sense defined above, then the interactions decay over some fixed time scale, $J(t)∼J_{0}e^{-|t|/\tau }$, and, at long times, the correlations also will decay exponentially. At the opposite extreme, if *x*(*t*) has effectively infinite dimensionality, then we can have $J(t)\approx J_{0}|t{|}^{-\alpha }$. Ising models with such power-law interactions are the subject of a large literature in statistical physics; the richest behaviors are at *α* = 2, where results presaged major developments in the renormalization group and topological phase transitions (59–62). It would be fascinating if these models emerged as effective descriptions of strongly non-Markovian sequences in animal behavior, as suggested recently (63).

## Generalization

In both the continuous Gaussian case and the discrete case, dimensionality can be measured through the problem of prediction. To make this more general, consider observations of behavior in a time window −T<t<T; for simplicity, I will keep the notation *x*(*t*) for the behavioral trajectory. Within each window, the trajectory x(t<0) defines the past $x_{\text{p}},\;x(t>0)$ defines the future $x_{\text{f}}$, and these are drawn from the joint probability distribution $P_{T}(x_{\text{p}},x_{\text{f}})$. To characterize the possibility of making predictions, we can measure the mutual information between past and future,

[22] $$I(x_{\text{past}};x_{\text{fut}})=\sum_{x_{\text{p}},x_{\text{f}}}P_{T}(x_{\text{p}},x_{\text{f}})\log\;\left[\frac{P_{T}(x_{\text{p}},x_{\text{f}})}{P_{T}(x_{\text{p}})P_{T}(x_{\text{f}})}\right].$$

This “predictive information” $I_{\text{pred}}(T)$ can have very different qualitative behaviors as *T* becomes large (64).

For a time series that can be captured by a finite-state Markov process, or more generally described by a finite correlation time, then $I_{\text{pred}}(T)$ is finite as T→∞. On the other hand, for Gaussian processes with correlation functions that decay as a power, as in Eq. **17**, the predictive information diverges logarithmically, $I_{\text{pred}}(T\to \infty )\propto \log\;T$, and similarly for discrete time series with power-law correlations.^‡^

In the example of a dynamical system with *D* variables, as in Eq. **16**, all the predictive power available will be realized if we can specify *D* numbers, which are the initial conditions for integrating the differential equations. Thus we consider smooth mappings of the past into *d* features,

[23] $$M_{d}:x_{\text{past}}\to \{F_{\mu }\},\;\;\;\mu =1,\;2,\;\cdots ,\;d.$$

For any choice of features, we can compute how much predictive information has been captured, and then we can maximize over the mapping, resulting in

[24] $$I_{\text{pred}}(T;d)=\underset{{}_{M_{d}}}{\max}I(\{F_{\mu }\};x_{\text{fut}}),$$

which is the maximum predictive information we can capture with *d* features in windows of duration *T*.

If the system truly is *D* dimensional, then *D* features of the past are sufficient to capture all of the available predictive information. This means that a plot of $I_{\text{pred}}(T;d)$ vs. *d* will saturate. To be precise, we are interested in what happens at large *T*, so we can define

[25] $$\underset{T\to \infty }{\lim\;}\frac{I_{\text{pred}}(T;d)}{I_{\text{pred}}(T)}=f(d).$$

If f(d≥D)=1, then we can write the analog of Eq. **14**,

[26] $$P[x_{\text{f}}|x_{\text{p}}]=P[x_{\text{f}}|\{F_{\mu }\}],$$

where the features $F_{\mu }$ now are more complex functions of the past. But there are only *D* of these features needed to make Eq. **26** true (μ=1, 2, ⋯, D), and so we conclude that the behavior has dimensionality *D*.

The equivalence of Eq. **26** to Eq. **14** immediately tells us that the general information theoretic definition of dimensionality agrees with the definition for Gaussian processes based on the spectrum of $K_{\text{pf}}$. In the Gaussian case, we see that the features $F_{\mu }$ are just linearly filtered versions of the past, as in Eq. **15**. The connection to the discussion of two-state variables is a bit more complicated, and exploits the equivalence to an internal or latent variable as described in *Appendix C*.

## Conclusion

The arguments here define the dimensionality of behavior as the minimum number of features of the past needed to make the maximally informative predictions about the future. As we consider pasts of longer duration, the dimensionality can grow, potentially without bound. The connection between dimensionality and prediction is familiar from the now classical literature on dynamical systems, which also reminds us that, in its most general form, any such definition runs into all the well-known difficulties of estimating dimensions from finite data (65). More useful is the result that, in some cases, estimating this predictive dimensionality reduces to analyzing the spectrum of a matrix.

## Data Availability

There are no data underlying this work.

## Footnotes

^*^In retroviruses, including HIV, reproduction occurs without proofreading. The dramatically accelerated pace of evolution in these viruses gives a glimpse of how different life would be if the transmission of genetic information depended on base pairing alone.

^†^See also the remarkable early work from Turner, who studied both insect behavior and neuroanatomy in the decades straddling 1900 (27).

^‡^If we observe a continuous variable in continuous time, then smoothness generates a formal divergence in the mutual information between past and future. Modern analyses of behavior typically begin with video data, with time in discrete frames, evading this problem. Alternatively, if measurements include a small amount of white noise, then the predictive information becomes finite even without discrete time steps. Thanks go to A. Frishman for emphasizing the need for care here.

## Appendix

### A. Past–Future Kernels, Explicitly.

We are interested in the behavior of the kernel *K* when the correlation function *C* is a sum of exponentials. As noted above, we need to be a little careful to make this problem well posed. If we monitor a continuous variable in continuous time, then continuity leads to infinite mutual information between $x(t^{-})$ and $x(t^{+})$. We can solve this either by assuming that observations are made at discrete ticks of a clock (as in video recordings) or by assuming that observations are made in a background of white noise. Here I will take the second approach.

The statement that the correlation function is a sum of exponentials, but measurements are in a background of white noise, means that the observed correlation function

[27] $$\langle x(t)x(0)\rangle \equiv C(t)=\sum_{\mu =1}^{M}A_{\mu }e^{-|t|/\tau_{\mu }}+N\delta (t),$$

where N is the strength of the noise. We want to construct the kernel *K*(*t*) that is the operator inverse to *C*, as in Eq. **10**. We recall that this can be done by passing to Fourier space,

[28] $$G(\omega )=\int dt\;e^{+i\omega t}C(t)$$

[29] $$K(t)=\int \frac{d\omega }{2\pi }e^{-i\omega t}\frac{1}{G(\omega )}.$$

From Eq. **27**, we can see that

[30] $$G(\omega )=\int dt\;e^{+i\omega t}\left[\sum_{\mu =1}^{M}A_{\mu }e^{-|t|/\tau_{\mu }}+N\delta (t)\right]$$

[31] $$=\sum_{\mu =1}^{M}\frac{2A_{\mu }\tau_{\mu }}{1+{\left(\omega \tau_{\mu }\right)}^{2}}+N.$$

Then, to find *K*(*t*), we invert and transform back, being careful to isolate the contribution of the white noise term,

[32] $$K(t)=\int \frac{d\omega }{2\pi }e^{-i\omega t}{\left[\sum_{\mu =1}^{M}\frac{2A_{\mu }\tau_{\mu }}{1+{\left(\omega \tau_{\mu }\right)}^{2}}+N\right]}^{-1}$$

[33] $$=\int \frac{d\omega }{2\pi }e^{-i\omega t}\left[\frac{1}{N}-\frac{P_{M-1}(\omega^{2})}{P_{M}(\omega^{2})}\right],$$

where

[34] $$P_{M-1}(\omega^{2})=\sum_{\mu =1}^{M}2A_{\mu }\tau_{\mu }\prod_{\nu \neq \mu }[1+{\left(\omega \tau_{\nu }\right)}^{2}]$$

is a *M* –1 st-order polynomial in $\omega^{2}$, and

[35] $$P_{M}(\omega^{2})=N\left(N\prod_{\mu =1}^{M}[1+{\left(\omega \tau_{\mu }\right)}^{2}]+P_{M-1}(\omega^{2})\right)$$

is a *M*th-order polynomial in $\omega^{2}$. Note that both polynomials have all real and positive coefficients.

We notice that $P_{M-1}(\omega^{2})/P_{M}(\omega^{2})$ vanishes at large |ω|, and $e^{-i\omega t}$ vanishes for values of *ω* with a large negative (positive) imaginary part if *t* > 0 (*t* < 0). This means that we can do the integral over *ω* in Eq. **33** by closing a contour in the complex plane. Then we can use the fact that

[36] $$P_{M}(\omega^{2})=B\prod_{\text{n}=1}^{M}\left(\omega^{2}-\omega_{\text{n}}^{2}\right),$$

where *B* is a constant and $\left\{\omega_{\text{n}}^{2}\right\}$ are the roots of the polynomial. The simplest case is where all $\omega_{\text{n}}^{2}$ are real, in which case they must be negative, and we can write $\omega_{\text{n}}=-i\lambda_{\text{n}}$, with $\lambda_{\text{n}}>0$. Then, for *t* > 0, we close the contour in the lower half plane, picking out the poles at $\omega =\omega_{\text{n}}$, while, for *t* < 0, we close the contour in the upper half plane, picking out the poles at $\omega =-\omega_{\text{n}}$. The result is that

[37] $$K(t)=\frac{1}{N}\delta (t)-\int \frac{d\omega }{2\pi }e^{-i\omega t}\frac{P_{M-1}(\omega^{2})}{P_{M}(\omega^{2})}$$

[38] $$=\frac{1}{N}\delta (t)-\frac{1}{B}\sum_{\text{n}}\frac{P_{M-1}(\omega^{2}=-\lambda_{\text{n}}^{2})}{2\lambda_{\text{n}}\prod_{\text{m}\neq \text{n}}\left(\lambda_{\text{m}}^{2}-\lambda_{\text{n}}^{2}\right)}e^{-\lambda_{\text{n}}|t|}.$$

If we look back at the derivation of Eq. **12**, we can see that a delta function term in *K*(*t*) does not contribute to coupling past and future. Thus K(t>0) collapses into the form of Eq. **13**,

[39] $$K(t+t\prime )=\sum_{\text{n}=1}^{M}a_{\text{n}}\phi_{\text{n}}(t)\phi_{\text{n}}(t\prime )$$

[40] $$a_{\text{n}}=\frac{1}{B}\frac{P_{M-1}(\omega^{2}=-\lambda_{\text{n}}^{2})}{2\lambda_{\text{n}}\prod_{\text{m}\neq \text{n}}\left(\lambda_{\text{m}}^{2}-\lambda_{\text{n}}^{2}\right)}$$

[41] $$\phi_{\text{n}}(t)=e^{-\lambda_{\text{n}}t},$$

and the dimensionality *D* = *M*, as we hoped: If the observed behavioral variable is Gaussian, and the correlation function can be written as the sum of *M* exponentials, then the system has underlying dimensionality *D* = *M*.

It is useful to work out the case *M* = 1. Then we have

[42] $$\langle x(t)x(0)\rangle \equiv C(t)=Ae^{-|t|/\tau_{c}}+N\delta (t),$$

And, after some algebra, we find

[43] $$K(t)=a_{1}e^{-\lambda_{1}|t|}$$

[44] $$\lambda_{1}=\frac{1}{\tau_{c}}\sqrt{1+2A\tau_{c}/N}.$$

It is useful to think more explicitly about the fact that we have embedded a correlated signal in the background of white noise, so we can write

[45] x(t)=y(t)+η(t)

[46] $$\langle y(t)y(t\prime )\rangle =Ae^{-|t-t\prime |/\tau_{c}}$$

[47] 〈η(t)η(t′)〉=Nδ(t−t′).

Only *y*(*t*) is predictable; the best predictions would be based on knowledge of y(t=0). One can then show that the best estimate of this quantity, given observations on the noisy *x*(*t*), is

[48] $$y_{\text{est}}(0)=\int_{0}^{\infty }dt\;K(t)x(-t),$$

with the same *K*(*t*) as in Eq. **43**. Thus, asking for the optimal prediction is the same as asking for the optimal separation of the predictable signal from the unpredictable noise (66).

### B. Details for Fig. 2.

To generate Fig. 2, I start with some assumed correlation function C(τ)≡〈x(t)x(t+τ)〉, sampled at discrete times $t_{\text{n}}=\text{n}\Delta t$. This defines a correlation matrix $C_{\text{nm}}=C(t_{\text{n}}-t_{\text{m}})$, and then the kernel is the inverse of this matrix. One row of the matrix $K_{\text{nm}}={\left(C^{-1}\right)}_{\text{nm}}$ provides a sampled version of the function K(t−t′), from which we can construct $K_{\text{pf}}=K(t+t\prime )$ from Eq. **12**. Note that ${\tilde{K}}_{\text{nm}}=K(t_{\text{n}}+t_{\text{m}})$ is a symmetric matrix, and, if we normalize the functions $\phi_{n}(t)$, then the coefficients $a_{\text{n}}$ in Eq. **13** are the eigenvalues of this matrix; these eigenvalues are plotted in Fig. 2.

As an example that should illustrate a finite dimensionality, consider

[49] $$C_{3}(\tau )=\frac{1}{3}\left[e^{-|\tau |/(4\Delta t)}+e^{-|\tau |/(32\Delta t)}+e^{-|\tau |/(256\Delta t)}\right].$$

To be a bit more realistic, I add measurement noise with an amplitude of 10%, independent in each time bin, so that $C_{\text{nm}}\to (1/1.01)C_{\text{nm}}+(0.01/1.01)\delta_{\text{nm}}$. Then the matrix *K_nm_* is computed by inverting $C_{\text{nm}}$ in a window of T=4,000Δt. The symmetric ${\tilde{K}}_{\text{nm}}$ is constructed in windows of T=100Δt or T=1000Δt, and then diagonalized to find the eigenvalues. As an example that could illustrate infinite dimensionality, I consider the power-law correlation function in Eq. **17**, with $t_{0}=\Delta t$ and α=1/2, then follow the same procedure as with *C*_3_.

### C. Interactions vs Latent Variables.

Models where the observed degrees of freedom depend on hidden or latent variables, but not directly on one another, are sometimes set in opposition to statistical physics models, where it is more natural to think about direct interactions. But, as explained also in the supplementary material of ref. 67, this dichotomy is incorrect. In fact, interacting models can be rewritten as models where individual element responds independently to some hidden or latent variables. As an example, the maximum entropy model in Eq. **21** can be rewritten exactly as a model in which the behavioral state at time *t* depends only on some internal variable *x*(*t*),

[50] $$P\left(\left\{\sigma_{t}\right\}\right)=\int Dx\;P[x(t)]\prod_{t}P(\sigma_{t}|x(t)+h),$$

[51] $$P(\sigma |x+h)=\frac{\exp\;\left[\sigma \cdot \left(x+h\right)\right]}{2\cosh\;(x+h)},$$

and the distribution of the internal variable is

[52] $$\begin{array}{l} P[x(t)]=\frac{1}{Z\prime }e^{-S\prime [x(t)]}\\ S\prime [x(t)]=\frac{1}{2}\sum_{t,t\prime }x(t)K(t-t\prime )x(t\prime )-\sum_{t}\ln\;\cosh\;\left(x(t)+h\right), \end{array}$$

where *K*(*t*) is the matrix inverse of the function *J*(*t*),

[53] $$\sum_{t\prime \prime }K(t-t\prime \prime )J(t\prime \prime -t\prime )=\delta_{tt\prime }.$$
